# Vitamin D deficiency contributes directly to the acute respiratory distress syndrome (ARDS)

**DOI:** 10.1136/thoraxjnl-2014-206680

**Published:** 2015-05-01

**Authors:** Rachel C A Dancer, Dhruv Parekh, Sian Lax, Vijay D'Souza, Shengxing Zheng, Chris R Bassford, Daniel Park, D G Bartis, Rahul Mahida, Alice M Turner, Elizabeth Sapey, Wenbin Wei, Babu Naidu, Paul M Stewart, William D Fraser, Kenneth B Christopher, Mark S Cooper, Fang Gao, David M Sansom, Adrian R Martineau, Gavin D Perkins, David R Thickett

**Affiliations:** 1 Centre for Translational Inflammation and Fibrosis Research, School of Clinical and Experimental Medicine, University of Birmingham, Birmingham, UK; 2 School of Cancer Sciences, University of Birmingham, Birmingham, UK; 3 Centre for Endocrinology, Diabetes and Metabolism, School of Clinical and Experimental Medicine, University of Birmingham, Birmingham, UK; 4 Norwich Medical School, University of East Anglia, Norwich, UK; 5 Renal Division, Brigham and Women's Hospital, Harvard Medical School, Boston, Massachusetts, USA; 6 Department of Medicine, Concord Medical School, University of Sydney, Sydney, New South Wales, Australia; 7 Institute of Immunity and Transplantation, University College London, London, UK; 8 Blizard Institute, Queen Mary University of London, London, UK; 9 Warwick Clinical Trials Unit, Warwick Medical School, University of Warwick, Coventry, UK

**Keywords:** ARDS, Innate Immunity

## Abstract

**Rationale:**

Vitamin D deficiency has been implicated as a pathogenic factor in sepsis and intensive therapy unit mortality but has not been assessed as a risk factor for acute respiratory distress syndrome (ARDS). Causality of these associations has never been demonstrated.

**Objectives:**

To determine if ARDS is associated with vitamin D deficiency in a clinical setting and to determine if vitamin D deficiency in experimental models of ARDS influences its severity.

**Methods:**

Human, murine and in vitro primary alveolar epithelial cell work were included in this study.

**Findings:**

Vitamin D deficiency (plasma 25(OH)D levels <50 nmol/L) was ubiquitous in patients with ARDS and present in the vast majority of patients at risk of developing ARDS following oesophagectomy. In a murine model of intratracheal lipopolysaccharide challenge, dietary-induced vitamin D deficiency resulted in exaggerated alveolar inflammation, epithelial damage and hypoxia. In vitro, vitamin D has trophic effects on primary human alveolar epithelial cells affecting >600 genes. In a clinical setting, pharmacological repletion of vitamin D prior to oesophagectomy reduced the observed changes of in vivo measurements of alveolar capillary damage seen in deficient patients.

**Conclusions:**

Vitamin D deficiency is common in people who develop ARDS. This deficiency of vitamin D appears to contribute to the development of the condition, and approaches to correct vitamin D deficiency in patients at risk of ARDS should be developed.

**Trial registration:**

UKCRN ID 11994.

**Key messages**

**What is the key question?**

- Is vitamin D deficiency a risk factor for the development of acute respiratory distress syndrome (ARDS)?

**What is the bottom line?**

- Patients with and at risk of ARDS are highly likely to be deficient, and severity of vitamin D deficiency relates to increased epithelial damage, the development of ARDS and survival.

**Why read on?**

- We present evidence that an easily treatable vitamin deficiency may increase the risk of ARDS in patients at risk.

## Introduction

Acute respiratory distress syndrome (ARDS) occurs due to either direct or indirect proinflammatory insults. However, only a proportion of at-risk patients develop ARDS, with research suggesting that genetic, age, social and other factors play a role in determining who develops ARDS.^1^

More than 1 billion people worldwide are believed to have vitamin D deficiency.^2^ Vitamin D has important functions besides bone and calcium homeostasis^3^ with cells of the innate and adaptive immune system responding to vitamin D. Vitamin D deficiency may therefore increase the risk of bacterial and viral infection.

Vitamin D deficiency is associated with an increased risk of intensive care admission and mortality in patients with pneumonia.^4^ Deficiency is common in critically ill patients and associated with adverse outcome.^3^ Gram-positive bacteria, invasive pneumococcal disease and meningococcal disease are more common when 25(OH)D_3_ levels are low.^5^ Recent data from an Austrian study in critically ill deficient patients suggests that when treatment with vitamin D is successful in raising levels >75 nmol/L there is a mortality benefit.^6^

Vitamin D may improve outcomes by reducing both local and systemic inflammatory responses as a result of modulating cytokine responses.^7^ In a mouse model of lethal endotoxaemia, survival post intravenous lipopolysaccharide (LPS) was significantly poorer in the vitamin D receptor knockout mice.^8^

Our aim was to define the prevalence and severity of vitamin D deficiency in patients with ARDS and to establish whether vitamin D deficiency is a risk factor for and/or a driver of the exaggerated and persistent inflammation that is a hallmark of ARDS. To achieve these aims, we employed translational clinical studies and in vitro primary cell work and murine models.

## Methods

### Patient cohorts

Patient cohort details are outlined in the online supplement but consisted of 52 patients with ARDS, 57 patients undergoing oesophagectomy (at risk of ARDS) and 8 patients undergoing oesophagectomy who had high-dose vitamin D supplementation prior to surgery.

*Patients with ARDS*: 52 patients who were recruited into the first beta agonist lung injury trial (BALTI-1) study^9^ and the translational sub-study of BALTI-2.^10^ There was no difference in age, sex, pre-enrolment Lung Injury score or acute physiology and chronic health evaluation (APACHE) II in these two groups of patients. Vitamin D levels were determined from ARDS patient plasma collected on the day of enrolment. In the oesophagectomy cohort, blood was collected on the day of the operation—pre any intervention.

Aetiology of ARDS is outlined in table 1. As these cohorts of patients were diagnosed prior to the Berlin criteria, throughout the paper we have used ARDS to indicate patients meeting criteria for acute lung injury or ARDS according to the definition of the American European Consensus Conference.^11^

**Table 1:** Comparison of demographics between ARDS and at-risk patients who were undergoing oesophagectomy

	ARDS (n=52)	At risk (n=65)	p Value
Male, n (%)	30 (57)	57 (87.6)	<0.001
Age (years), mean (SD)	61.3 (16.7)	62.8 (10.8)	0.560
Predisposing condition, n (%)	Pneumonia 18 (35) Other sepsis 24 (46) Aortic aneurysm repair 3 (6) Chest trauma 2 (3.8) Pancreatitis 1 (1.9) Transfusion-related lung injury 1 (1.9) Other 3 (6)	Oesophagectomy 65 (100)	n/a
LIS, median (IQR)	2.75 (2.50–3.19)	1.5 (1.0–2.0)	<0.001
APACHE II median (IQR)	24 (19–28)	12 (8–14)	<0.001
Worst P/F ratio during admission, mean (SD)	14.9 (5.2)	31.5 (9.9)	<0.001
Hospital survival, n (%)	16 (30.8)	62 (95.4)	<0.001
Length of hospital stay for survivors (days), median (IQR)	35 (16–49)	17 (10–28)	0.025

Statistical tests used are χ^2^ t test where data is normally distributed and Kruskal–Wallis for non-parametric data.

APACHE, acute physiology and chronic health evaluation; ratio of arterial oxygen tension to the fraction of inspired oxygen (Pao_2_:Fio_2_), arterial oxygen tension: fractional inspired oxygen; ARDS, acute respiratory distress syndrome; LIS, lung injury score.

### Pulse Contour Cardiac Output Monitoring (PiCCO)

Extravascular lung water (EVLW) was measured using the single-indicator transpulmonary thermodilution system (PiCCO-II; Pulsion) as described previously.^12^ In our study, the coefficient of variance for this system was <7% for all parameters.

*Vitamin D status*: 25(OH)D_3_ was measured by tandem mass spectroscopy using appropriate Vitamin D External Quality Assessment Scheme control. 1,25(OH)_2_D and vitamin D binding protein (VDBP) were measured by ELISA. Definition of vitamin D status is controversial, with different figures used throughout the literature, but for this study we have considered plasma 25-OH vitamin D_3_ levels <50 nmol/L as deficient and levels <20 nmol/L as severe deficiency.^13^ ^14^ In addition, patients in the at-risk cohort with 25(OH) vitamin D_3_ <20 nmol/L had significantly lower 1,25(OH)_2_ vitamin D than patients with higher levels (<20 nmol/L 74 pmol/L vs >20 nmol/L 90 pmol/L, p=0.029).

*Murine cytokines* were measured by multiplex array (R&D, UK) or ELISA as per the manufacturer's instruction.

### ATII cell isolation and culture

ATII cells were extracted from lung resection specimens according to the methods described previously^15^ (see online supplementary material).

*Microarray analysis* is outlined in the online supplementary material.

*Wound repair, proliferation and cell death assays* were performed as described previously.^16^

### Mouse methods

Wild-type (WT) C57Bl/6 mice were obtained from Harlan UK, Oxford, UK, and maintained at BMSU, Birmingham University, UK. Once weaned, vitamin D deficiency was induced in WT pups by feeding them a vitamin D-deficient chow (Harlan, USA) for 6 weeks pre-intra-tracheal (IT) LPS. 25(OH)-vitamin D was assessed by direct ELISA (ImmunDiagnostik, Germany). The LPS challenge model was performed as described previously.^17^ Briefly, mice are anaesthetised and 50 µg LPS (Sigma, UK) instilled by IT route as a model of direct lung injury. Mice were sacrificed at neutrophilic peak, 48 h post-LPS instillation, and bronchoalveolar lavage (BAL) performed with two washes of 0.6 mL phosphate buffered saline (PBS)/EDTA (200 nM) installations to determine the local effects on inflammation. Untreated controls were also analysed to determine lung parameters in vitamin D-deficient mice. BAL fluid (BALF) was assessed for cellular inflammation by cell count, neutrophilia and neutrophil apoptosis (flow cytometry), markers of epithelial damage BALF receptor for glycosylated endpoints (BALF RAGE), protein permeability index (ratio of BALF protein:plasma protein) as well as cytokines by luminex array (R&D systems, UK). Results represent mice from three separate experiments with at least four replicates per group. Oxygen saturations were measured at 48 h post-LPS and compared with WT mice given PBS by MouseOx II Plus (n=8 for each condition). All experiments were performed in accordance with UK laws with approval of local animal ethics committee.

*Statistics* Data were analysed using SPSS for Windows 16.0 (SPSS, Chicago, Illinois, USA). Data were tested for normality and analysed by unpaired t tests or Mann–Whitney U test. Data are expressed as mean (SD) unless otherwise indicated. A χ^2^ or Fisher’s exact test was used to compare proportions.

To test the hypothesis that low 25(OH)D levels are associated with the development of ARDS in the at-risk oesophagectomy cohort (N=65), we performed multivariable logistic regression with the exposure of interest being 25(OH)D_3_ level <20 nmol/L and ARDS as the outcome. Adjusted ORs were estimated by multivariable logistic regression models with inclusion of covariate terms chosen, a priori, thought to be plausibly associated with both 25(OH)D_3_ level and ARDS in the oesophagectomy patient cohort. We sought to build a parsimonious model that did not unnecessarily adjust for covariates that did not affect bias or the causal relation between exposure and outcome. Model calibration was assessed using the Hosmer–Lemeshow (HL) χ^2^ goodness-of-fit test and the accompanying p value. Bayesian information criterion and Akaike information criterion were also used to determine global model fit. Covariates included in the logistic regression model were age, gender, diagnosis, staging and pack-years smoked. The discriminatory ability for ARDS was quantified using the c-statistic. In all analyses, p values are two-tailed and values below 0.05 were considered statistically significant.

## Results

### Plasma vitamin D status in patients with or at risk of ARDS

**Figure 1:**
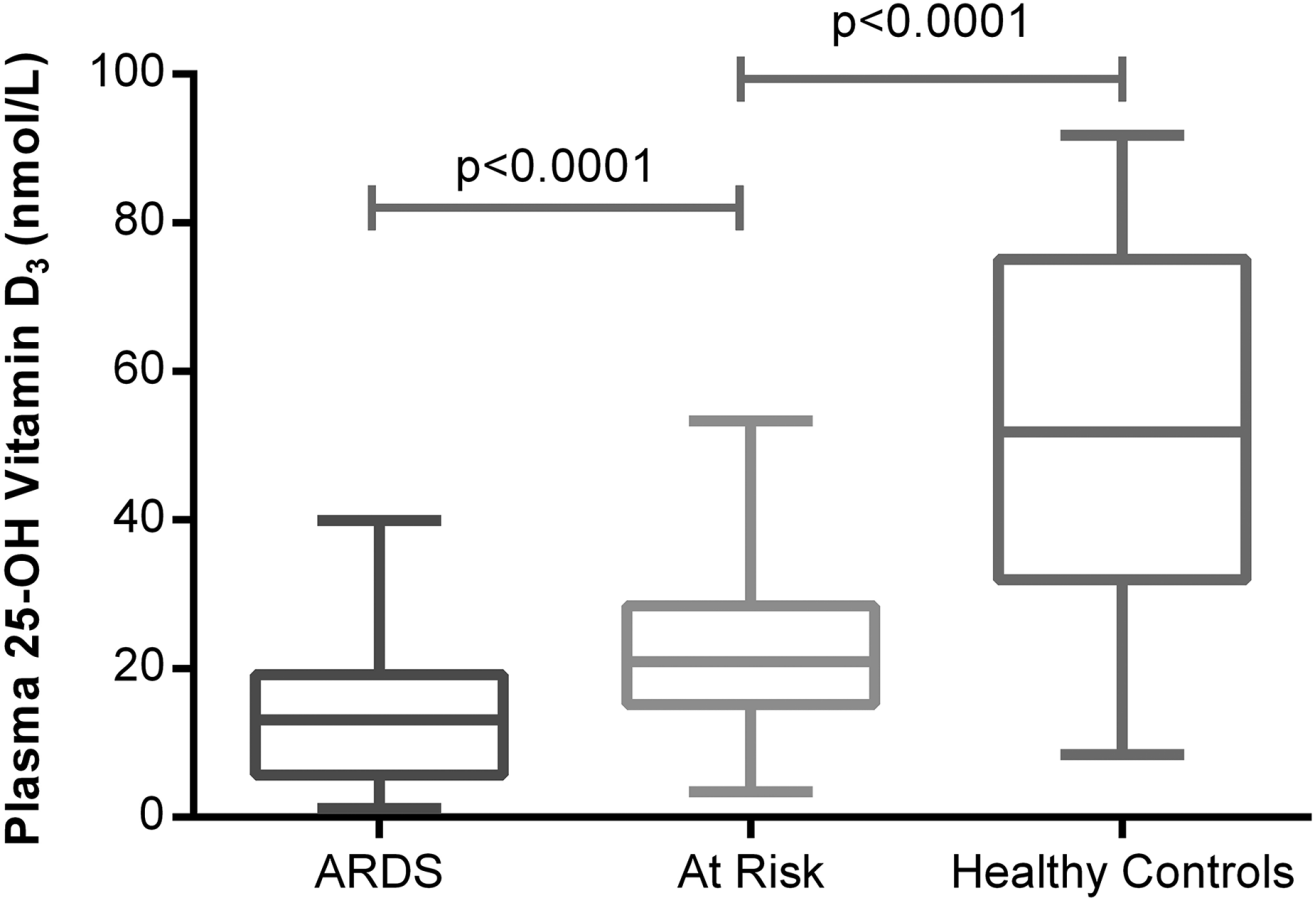
Plasma 25(OH)D_3_ levels in acute respiratory distress syndrome (ARDS) versus at risk and normal controls. The *horizontal bar* represents the median, and the *boxes* represent IQRs. *Vertical lines* show minimum–maximum range. Fifty-two patients with ARDS, 57 at-risk patients undergoing oesophagectomy, 18 healthy controls.

Patients with ARDS (100%) were vitamin D-deficient (plasma 25(OH)D_3_ <50 nmol/L). In total, 55 (96%) out of 57 unsupplemented oesophagectomy patients at risk of ARDS were deficient preoperatively but levels were higher than in patients with ARDS. Both patients at risk and patients with ARDS had significantly lower levels of 25(OH)D_3_ than normal controls (see figure 1). Demographics of patients groups based on vitamin D levels are illustrated in table 2.

**Table 2:** Comparison of demographics between patients with severe deficiency, moderate deficiency and vitamin D supplemented at-risk patients undergoing oesophagectomy

	Patients with severe 25-OH vitamin D_3_ deficiency (n=25)	Patients with moderate 25-OH vitamin D_3_ deficiency (n=32)	Patients who received vitamin D supplementation (n=8)	p Value
Male, n (%)	21 (84)	28 (87.5)	8 (100)	0.706
Age, years median (IQR)	60.0 (52.0–68.5)	65.5 (54.5–72.0)	68.0 (63.8–71.3)	0.122
BMI median (IQR)	24.3 (20.6–27.9)	25.4 (21.7–28.3)	24.1 (22.0–26.6)	0.698
ASA median (IQR)	2.0 (2.0–2.0)	2.0 (2.0–2.0)	2.0 (2.0–2.75)	0.950
Postoperative P/F ratio	41.0 (34.3–53.3)	39.8 (32.0–52.0)	47.8 (39.4–50.8)	0.476
Preoperative plasma 25-OH vitamin D_3_ level (nmol/L) median (IQR)	13.7 (10.9–16.7)	27.6 (22.5–34.9)	66.9 (42.5–92.6)	<0.001

All p values shown are Kruskal–Wallis tests from SPSS.

ASA, American Society of Anaesthesiologists physical status classification system; BMI, body mass index.

**Figure 2:**
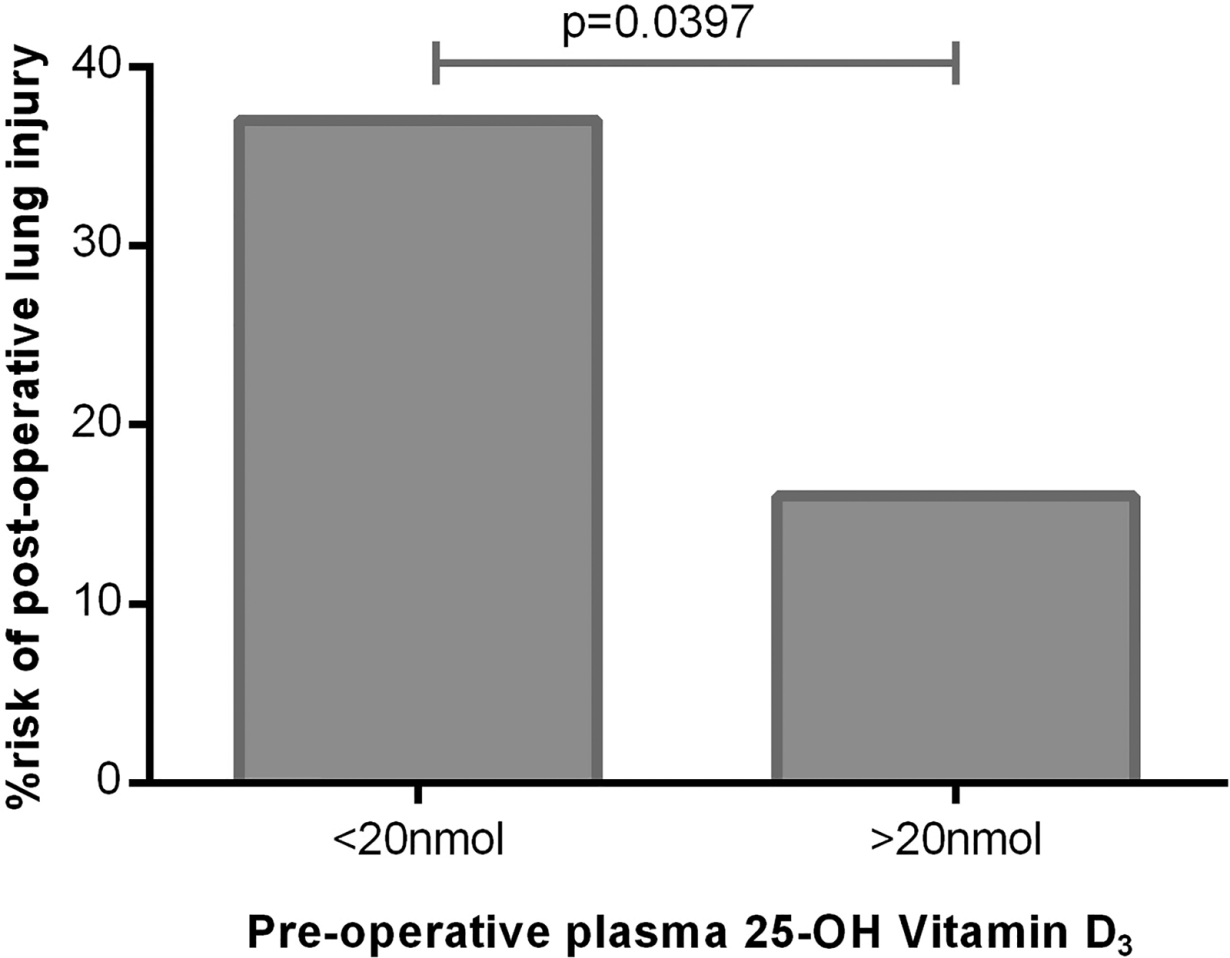
Risk of postoperative acute respiratory distress syndrome in severe 25(OH)D_3_ deficiency versus less severe deficiency. Severe deficiency (n=25), less severe (n=32).

In at-risk patients, preoperative median plasma levels of 25(OH)D_3_ were significantly lower in those patients who were ventilated with ARDS postoperatively (ARDS 16.97 nmol/L vs no ARDS 25.46 nmol/L, p=0.014). Oesophagectomy patients with severe vitamin D deficiency (plasma 25(OH)D_3_ <20 nmol/L) had a 37.5% risk of postoperative lung injury as opposed to a 15% risk with vitamin D levels >20 nmol/L (figure 2).

In the at-risk oesophagectomy cohort, preoperative vitamin D status was the only measure to have a significant difference in oesophagectomy patients who develop lung injury postoperatively (see table 3).

**Table 3:** Univariate analysis of predictors of postoperative ARDS in patients undergoing oesophagectomy

	Patients with ARDS (n=15)	Patients without ARDS (n=50)	p Value
Male, n (%)	14 (93)	43 (86)	0.448
Age (years), median (IQR)	61 (53–66)	66 (56–71)	0.304
BMI (kg/cm^2^), median (IQR)	25.2 (23.9–29.1)	24.8 (21.6–28.1)	0.460
FEV_1_ (L), median (IQR)	2.69 (2.28–3.50)	2.85 (2.38–3.3)	0.901
FVC (L), median (IQR)	4.3 (3.4–5.2)	4.1 (3.5–4.7)	0.450
Tumour type=adenocarcinoma, n (%)	12 (80)	35 (70)	0.511
Tumour stage, n (%)*			
T2	4 (27)	12 (24)	0.865
T3	11 (73)	37 (76)	
N0	3 (20)	12 (24)	0.719
N1–2	12 (80)	37 (76)	
Smoker, n (%)			
Current	6 (40)	11 (22)	0.304
Former	7 (47)	34 (68)	
Never	2 (13)	5 (10)	
Pack-years, median (IQR)	30 (20–40)	30 (15–45)	0.740
Plasma 25-OH vitamin D_3_ (nmol/L), Median (IQR)	16.97 (12.98–22.46)	25.46 (17.35–39.77)	0.014
Plasma 1,25(OH)^2^ vitamin D (pmol/L), median (IQR)	68 (47–91)	89 (76–109)	0.007

Only preoperative 25(OH)D_3_ and 1,25(OH)_2_D vitamin D were significantly different in univariate analysis.

*No lung function available for seven patients, pack-years not available for two patients. One patient (without lung injury) had a benign tumour—not included in staging data.

ARDS, acute respiratory distress syndrome; BMI, body mass index.

The odds of ARDS in patients with 25(OH)D_3_ <20 nmol/L was 3.5-fold that of patients with 25(OH)D_3_ ≥20 nmol/L (OR=3.5 (95% CI 1.06 to 11.6; p=0.040)). Following adjustment for gender, age, diagnosis, staging data, and pack-years, patients with 25(OH)D_3_ <20 nmol/L had a 4.2-fold higher odds of ARDS than patients with 25(OH)D <20 nmol/L (OR=4.2 (95% CI 1.13 to 15.9; p=0.032)). The adjusted model showed good calibration (HL χ^2^ 11.10, p=0.20) and discrimination for ARDS (area under the curve 0.73). When 25(OH)D was analysed with logistic regression as a continuous exposure in 1 nmol/L increments, the odds of ARDS decreases by 17% for every 1 nmol/L decrease in 25(OH)D (OR 0.83 (95% CI 0.69 to 0.98; p=0.033)), adjusted for age, gender, diagnosis, staging and pack-years smoked.

### Plasma levels of 1,25(OH)_2_D are lowest in ITU non-survivors

**Figure 3:**
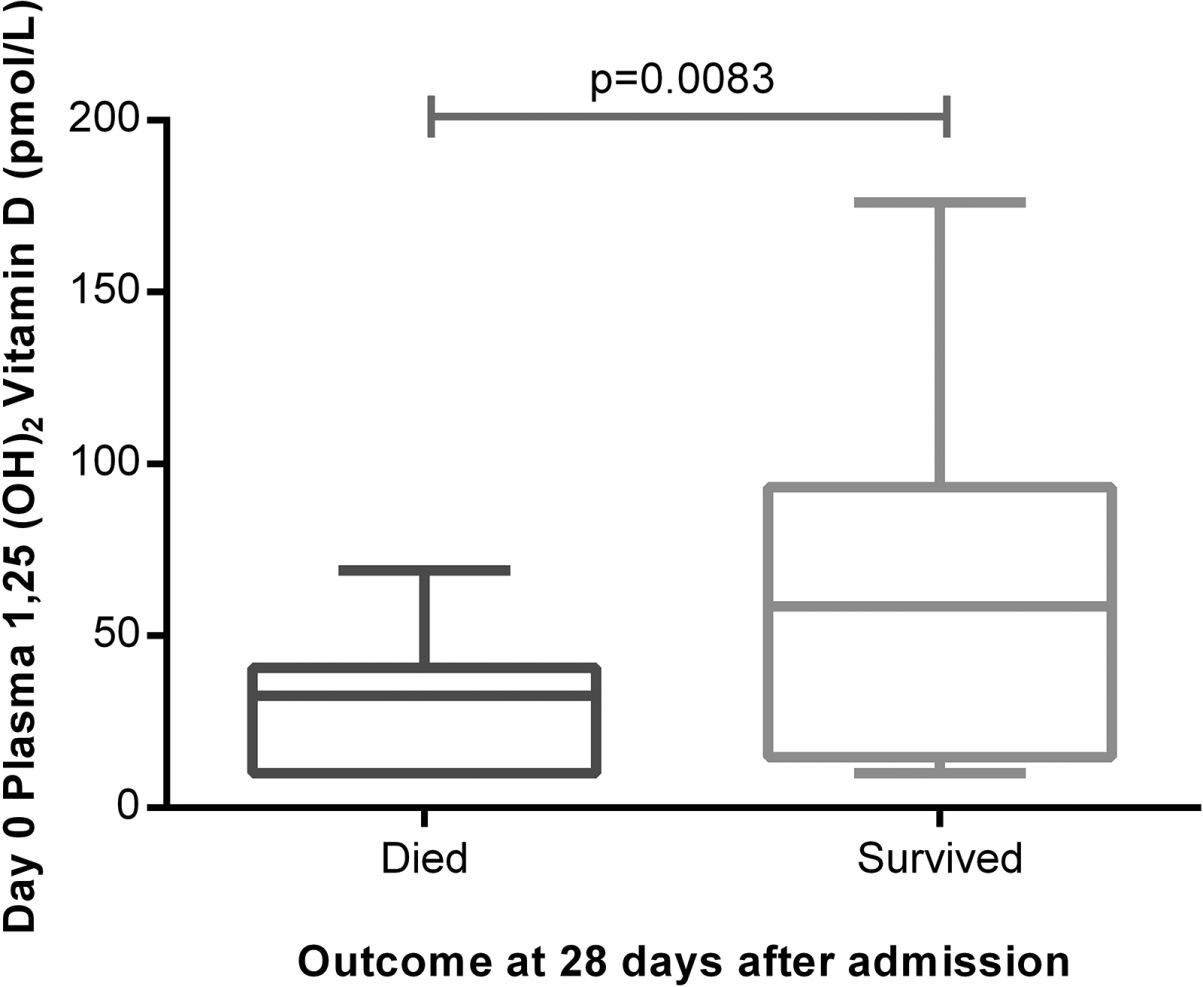
Plasma 1,25(OH)_2_D was significantly higher in patients with acute respiratory distress syndrome who survived at least 28 days following admission than those who died. The *horizontal bar* represents the median, and the *boxes* represent IQRs. *Vertical lines* show minimum–maximum range. Died (n=32), survived (n=20).

Median plasma 1,25(OH)_2_D levels were also significantly lower in patients with ARDS (35.5 pmol/L) than in at-risk patients (85 pmol/L, p=0.0001). Plasma 1,25(OH)_2_D was lower at admission to intensive therapy unit (ITU) in patients who died than survivors (figure 3). Plasma levels of 1,25(OH)_2_D were lower in oesophagectomy patients at risk of ARDS who subsequently went on to be ventilated for ARDS (68 pmol/L (IQR 47–91)) than those who did not get postoperative ARDS (89 pmol/L (IQR 76–109), p=0.007).

### Plasma VDBP levels are lower in patients with ARDS.

**Figure 4:**
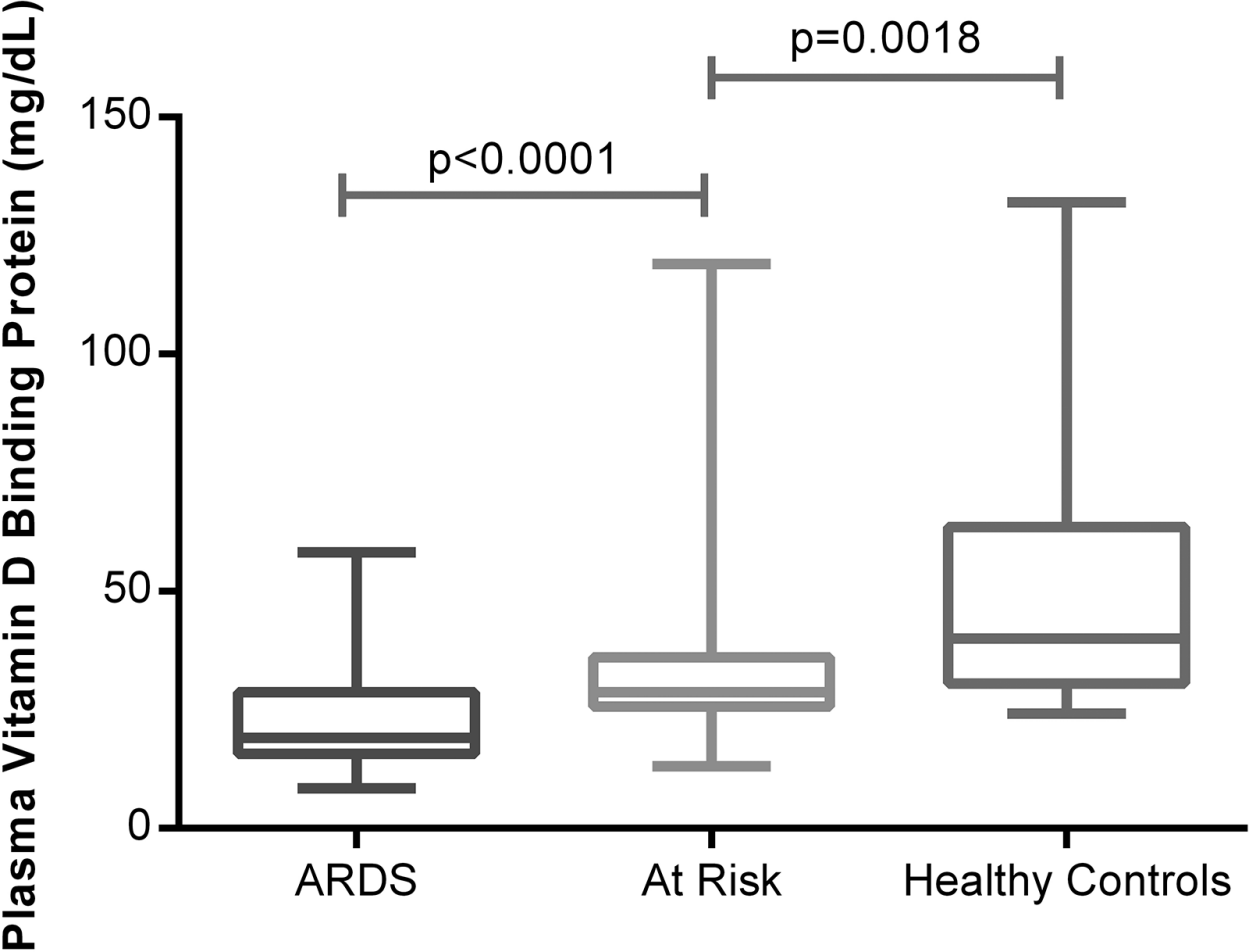
Plasma vitamin D binding protein measured by ELISA in acute respiratory distress syndrome (ARDS) versus at risk and normal controls. Fifty-two patients with ARDS, 57 at-risk patients undergoing oesophagectomy, 18 healthy controls.

25(OH)D_3_ circulates tightly bound to the VDBP (also known as Gc-actin).^18^ VDBP levels were 40 mg/dL in normal controls, 19 mg/dL in ARDS and 28.7 mg/mL in the at-risk patients at the beginning of oesophagectomy (figure 4).

### Vitamin D levels and perioperative changes in epithelial integrity in patients at risk of ARDS

We measured perioperative changes in an in vivo measure of the integrity of the alveolar–capillary barrier, namely EVLW accumulation (extravascular lung water index (EVLWI)) and pulmonary vascular permeability index (PVPI) using a PiCCO_2_ catheter^19–22^ and related this to the patient's vitamin D status.

**Figure 5:**
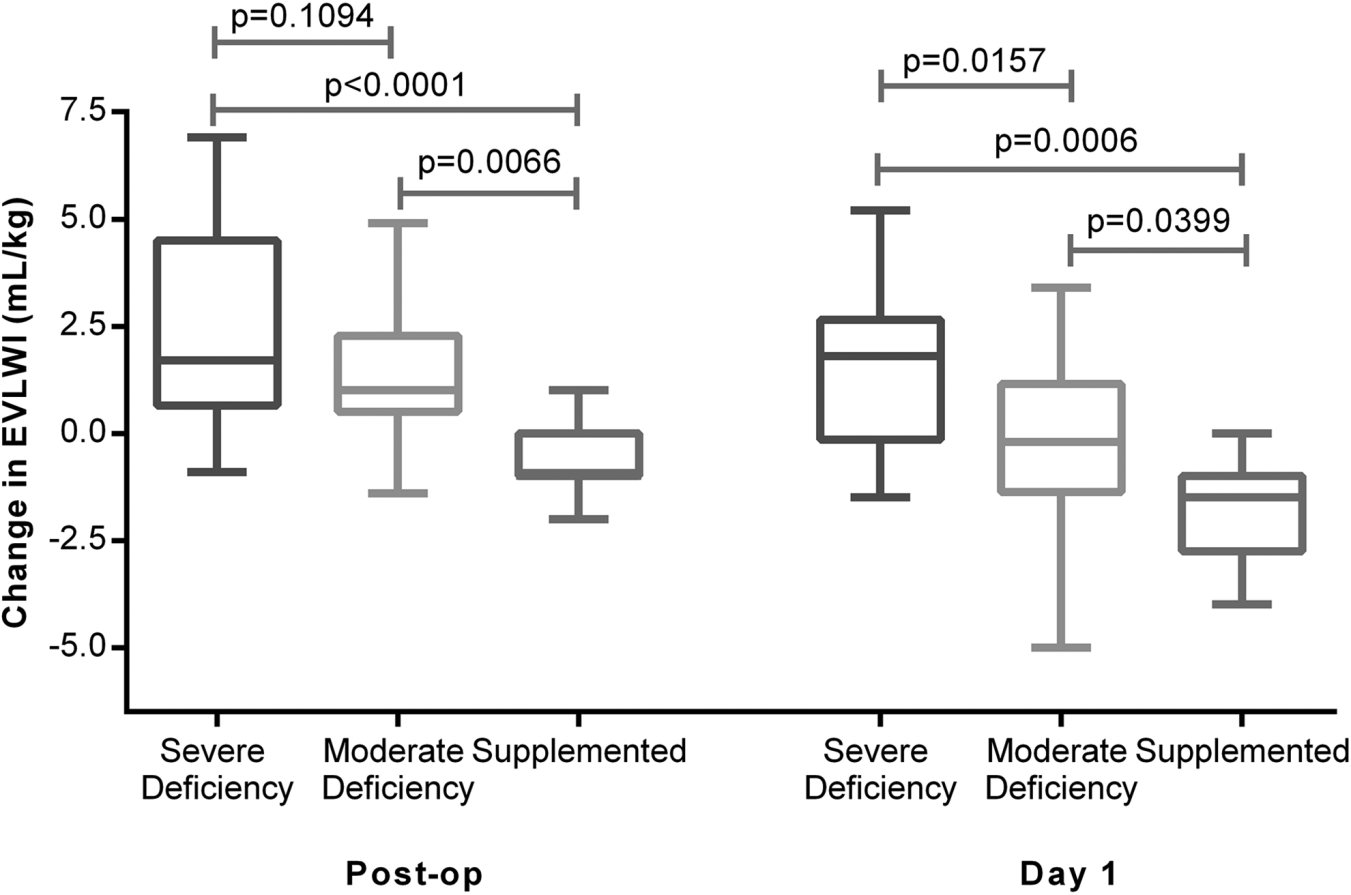
Changes in extravascular lung water index (EVLWI) at the end of oesophagectomy and on the morning of postoperative day 1. EVLWI was measured using Pulse Contour Cardiac Output Monitoring II catheter at the end of the operation and on the morning after the operation (day 1). Severe deficient (n=25), moderate (n=32) and supplemented (n=8).

**Figure 6:**
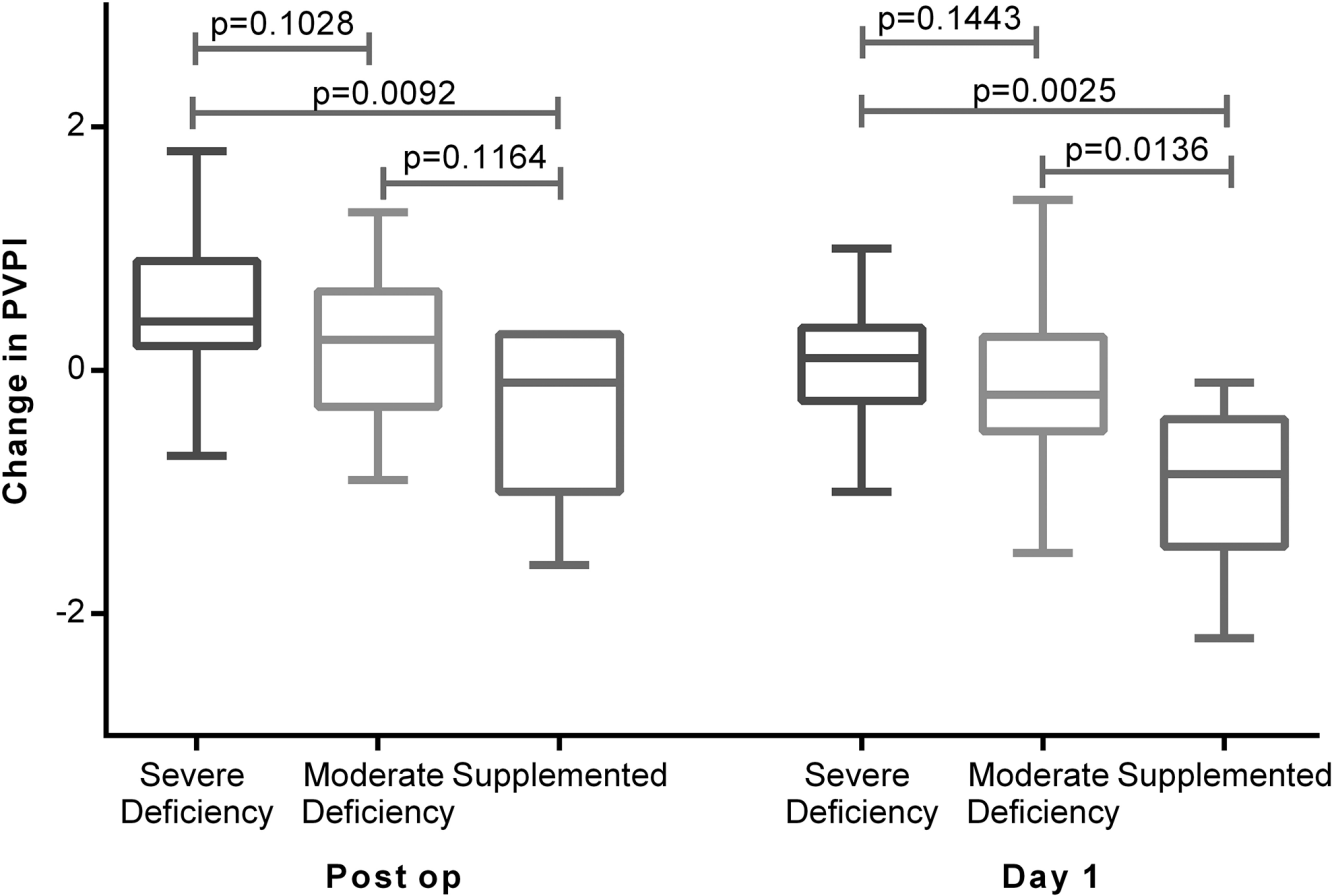
Changes in Pulse Contour Cardiac Output Monitoring pulmonary vascular permeability index (PVPI) at the end of oesophagectomy and the morning of postoperative day 1. Severe deficient (n=25), moderate (n=32) and supplemented (n=8).

Severe vitamin D deficiency (25-(OH)D_3_ <20 nmol/L) was associated with an increased accumulation of EVLW as assessed by PiCCO EVLWI and evidence of increases of PVPI, a marker of alveolar capillary permeability. Patients supplemented with vitamin D prior to oesophagectomy had significantly reduced changes in PiCCO EVLWI and PVPI than unsupplemented patients (figures 5 and 6).

### Vitamin D deficiency is a determinant of inflammation and epithelial injury in the intratracheal LPS murine model of ALI/ARDS

**Figure 7:**
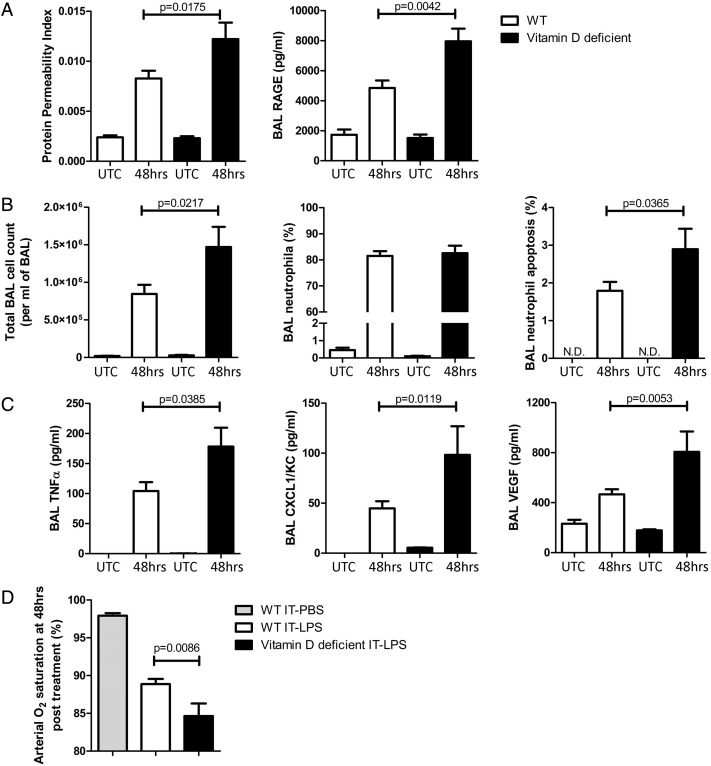
Lung injury and inflammation was significantly higher in vitamin D-deficient mice compared with wild-type (WT) following intra-tracheal (IT)-lipopolysaccharide (LPS). Levels of tumour necrosis factor-α and CXCL1/KC in UTCs were below the detection threshold of the assays performed. UTC, untreated control; N.D., not detected.

We studied the response to 50 µg of IT LPS in WT or mice made vitamin D deficient by dietary manipulation. Deficient mice were fed a vitamin D-free diet for 6 weeks and had median plasma vitamin 25(OH)D_3_ levels of 8 nmol/L (SEM 1.15 nmol/L) vs 42 nmol/L (SEM 2.17 nmol/L) in WT (p=0.001). Untreated vitamin D-deficient mice had no observed lung damage or inflammation (figure 7, and data not shown).

Following LPS challenge, vitamin D-deficient mice had increased evidence of alveolar epithelial damage as measured by BALF RAGE and BALF permeability index (figure 7A). Cellular inflammation and neutrophil apoptosis in BALF were also elevated in vitamin D-deficient mice, along with release of proinflammatory cytokines tumour necrosis factor-α, CXCL1/KC and vascular endothelial growth factor (figure 7B and C, respectively). These changes resulted in significantly lower oxygen saturation as measured by pulse oximetry (figure 7C), which we have previously demonstrated as a physiological measure of murine lung function.^15^

### Vitamin D is trophic for alveolar epithelial cells in vitro

ATII cells were treated with 100 nmol/L of 25(OH)D_3_ for 24 h. Microarray analysis revealed that vitamin D treatment caused a sustained activation or inhibition of 660 genes that included pathways involved in vitamin metabolism as well as regulators of cell growth, differentiation and response to wounding (GEOSET record GSE46749). The online supplementary table SA and SB outline the top 25 genes up-regulated and down-regulated by vitamin D and a heat map illustrating up-regulated and down-regulated genes. Table 4 outlines the biological processes and molecular functions modified by vitamin D treatment. Several of the identified pathways had significant relevance to proliferation, wound repair and apoptosis, so we tested the functional effects of vitamin D upon these important repair/protective processes.

**Table 4:** List of 30 statistically significant gene ontology (GO) terms implicated by differential expression of genes in day 3 epithelial (type II like) cells treated with vitamin D_3_ 100 nM relative to untreated cells

GO	Annotated genes	Total	p Value
	587	2230	
Immune response	48	76	0.00000
Immune system process	58	103	0.00000
Cytokine activity	25	36	0.00001
Extracellular process	45	86	0.00003
Signal transducer activity	75	173	0.00008
Molecular transducer activity	75	173	0.00008
Plasma membrane	133	356	0.00013
DNA replication	20	29	0.00015
Receptor activity	57	124	0.00015
Defence response	42	83	0.00015
Monoxygenase activity	12	13	0.00018
ATPase activity, coupled to transmembrane movement of substances	6	107	(0.00018)
Primary active transmembrane transporter activity	6	107	(0.00018)
Hydrolase activity, acting on acid anhydrides, catalysing transmembrane movement of substances	6	107	(0.00018)
P-P-bond-hydrolysis-driven transmembrane transporter activity	6	107	(0.00018)
ATPase activity, coupled to movement of substances	6	107	(0.00018)
Cell surface receptor linked signal transduction	69	162	0.00025
Chemotaxis	14	17	0.00027
Taxis	14	17	0.00027
Response to external stimulus	50	108	0.00030
Response to wounding	38	76	0.00043
Heme binding	12	14	0.00060
Tetrapyrrole binding	12	14	0.00060
Cellular biosynthetic process	15	145	(0.00105)
Biosynthetic process	29	214	(0.00121)
Extracellular region part	58	137	0.00168
Nucleotide biosynthetic process	1	61	(0.00168)
Cell cycle process	44	97	0.00208
Nucleobase-containing small molecule metabolic process	3	69	(0.00412)
Nucleotide metabolic process	3	68	(0.00466)

p values of underrepresented GO terms are denoted in parentheses**.**

### Effect of physiologically relevant doses of 25(OH)D_3_ upon primary human alveolar type II cells

**Figure 8:**
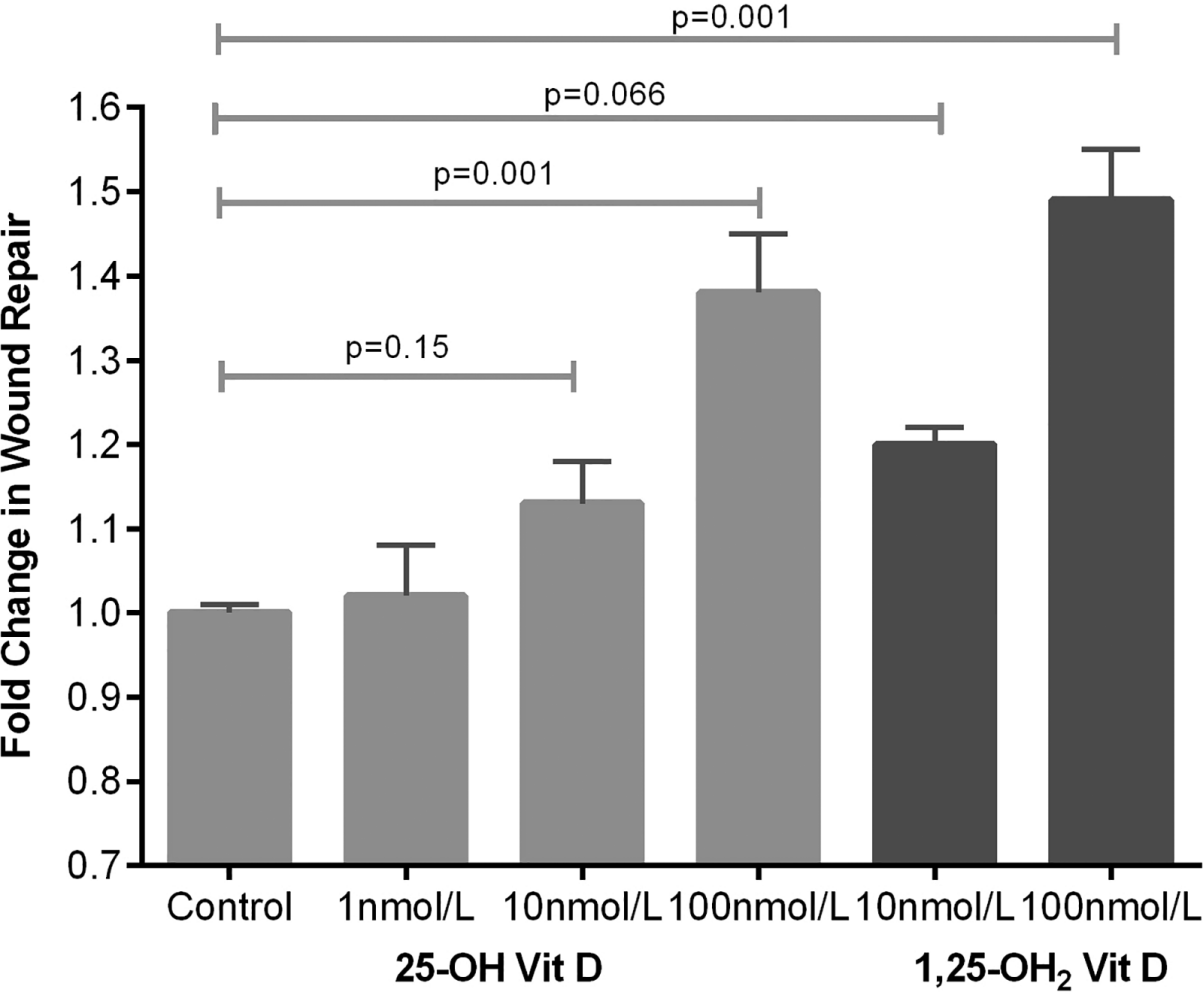
Scratch wound repair response of primary human alveolar type II cells to 25(OH)D_3_. Wound area after 24 h was compared with baseline and expressed as fold change in wound area. Data represents experiments using cells from six separate lung resection specimens. Analysis of variance p=0.001.

**Figure 9:**
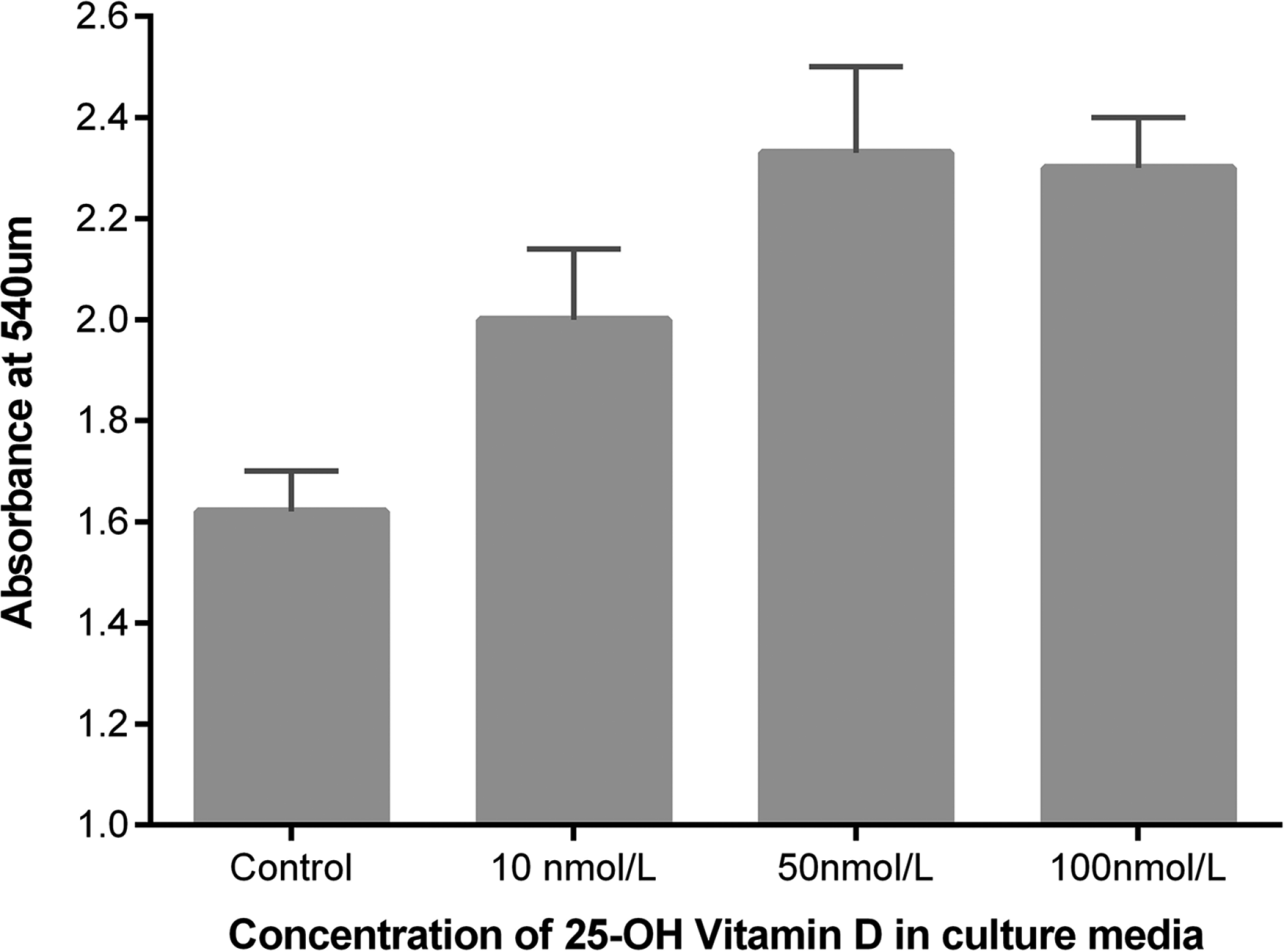
Proliferation of primary human ATII cells in response to physiological doses of 25(OH)D_3_ by bromodeoxyuridine incorporation. Experiments were performed using cells from four donors. Analysis of variance p=0.001.

**Figure 10:**
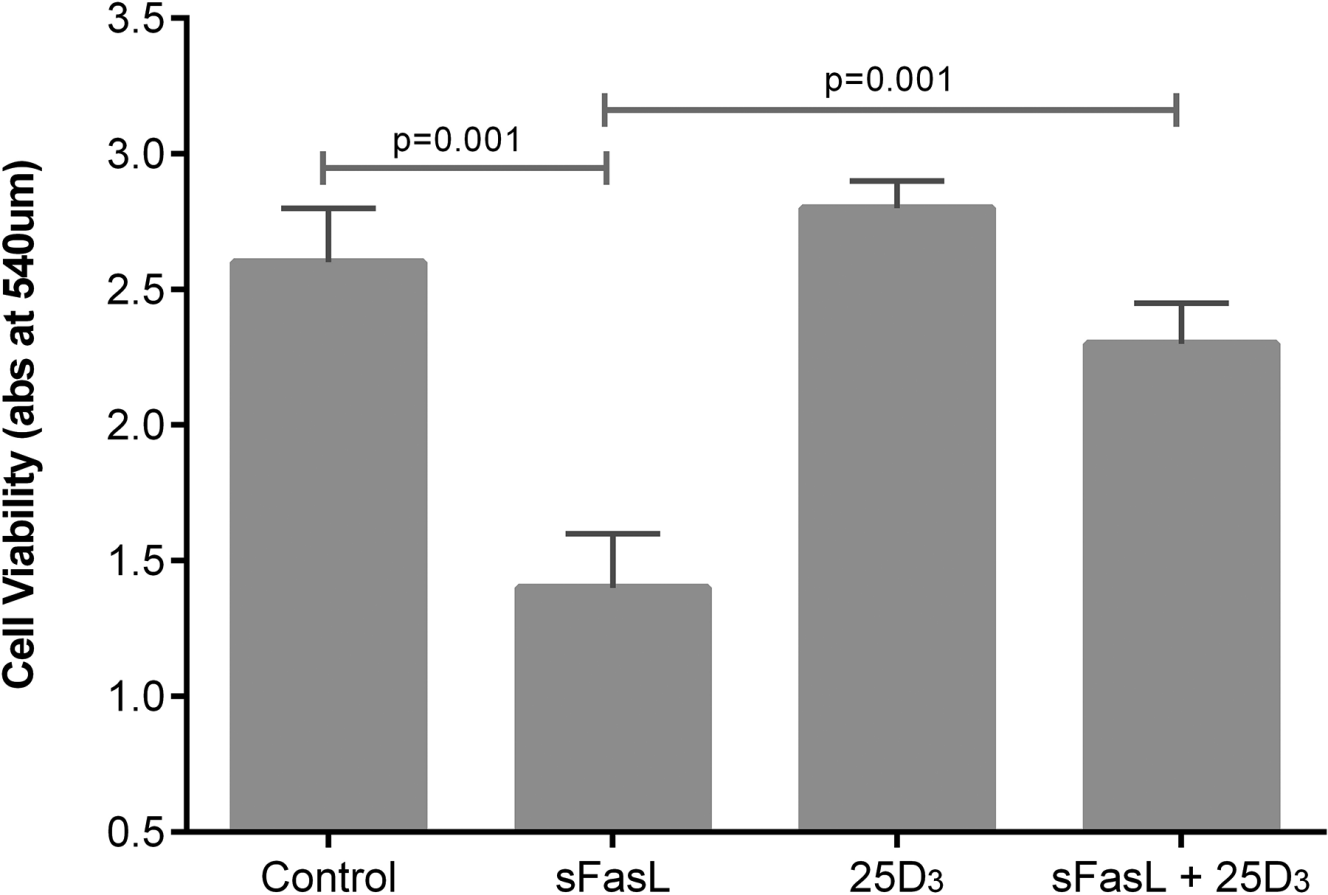
Cellular response to soluble Fas ligand (sFasL) 10 ng/mL induced cell death. Experiments were performed using ATII cells from four donors. 100 nmol/L 25(OH)D_3_ was added at the time of addition of sFasL.

25(OH)D_3_ at physiologically relevant concentrations stimulated scratch wound repair, cell proliferation and attenuated soluble Fas ligand (sFasL)-mediated cell death (see figures 8–10).

## Discussion

We have assessed the vitamin D status of a large cohort of patients with ARDS and a well-characterised group of patients at risk of ARDS, namely patients undergoing oesophagectomy. In ARDS cases, vitamin D deficiency was ubiquitous. Survivors of ARDS had significantly higher levels of vitamin D than non-survivors.

Our finding of a 30% reduction in VDBP in patients with ARDS supports a role for either reduced production or increased losses as an explanation for some of the degree of deficiency seen. Equally the low observed levels of circulating 1,25(OH)_2_D in patients with ARDS suggests a problem with renal metabolism as this is probably the major source of circulating 1,25(OH)_2_D.^23^

Several studies have suggested that vitamin D deficiency may be a risk factor for adverse outcome in pneumonia^24^ and lower respiratory tract infections in neonates.^25^ Other studies have suggested patients with sepsis have significant vitamin D deficiency.^26^ ^27^ Our data suggests in the high-risk oesophagectomy group that vitamin D status is also a pre-existing risk factor for ARDS—especially when deficiency is severe. Patients undergoing oesophagectomy with severe preoperative vitamin D deficiency had greater risk of postoperative ARDS and increases in PiCCO measures of alveolar permeability than those with less severe deficiency.

In our animal model of LPS-induced lung injury, vitamin D deficiency was associated with greater BALF cellular inflammation and cytokine release at 48 h. Increased epithelial damage and accumulation of apoptotic neutrophils was also evident. Mice that were vitamin D deficient became more hypoxic, suggesting physiologically worse lung injury.

Our animal data is in keeping with recently published data in hamsters treated with LPS.^28^ In contrast, Klaff *et al* found reduced neutrophil chemotactic potential to the chemokine KC ex vivo in mice deficient in vitamin D but no differences in LPS-induced BALF neutrophilia. They used a 72 h time point and a much lower dose of LPS (2.5 µg), which we suggest accounts for the differences with our study.

In both our human at-risk patients and the murine model, we have demonstrated evidence of increased permeability of the alveolar capillary barrier in response to one lung ventilation (EVLWI and PVPI) and LPS challenge respectively (PPI) when severe deficiency is present, suggesting that vitamin D might have protective effects on the alveolar epithelium as well as being anti-inflammatory. 1,25(OH)_2_D has been shown to induce DNA incorporation in human alveolar type II cells,^29^ but the effects of physiologically relevant doses of 25(OH)D have not been addressed upon type II cells previously. To address whether 25(OH)D_3_ has effects on ATII cells, we demonstrated considerable functional activity of a physiological dose of 25(OH)D_3_ by microarray analysis. Physiologically relevant doses of 25(OH)D_3_ stimulated wound repair, cellular proliferation and reduced sFasL-induced cell death. These in vitro experiments suggest that 25(OH)D_3_ may play a trophic role on adult human alveolar epithelial cells.

This study has limitations. First, although we obtained blood from patients with ARDS as soon as possible following admission to ITU, we are unable to be sure that levels of 25(OH)D_3_ were low prior to the development of ARDS in that cohort or whether levels fall because of the development of ARDS. Second, our at-risk data from oesophagectomy patients has to be divided into severe deficiency and moderate deficiency because of the severity of vitamin D deficiency observed in that patient group. Third, our data in oesophagectomy patients’ needs validating in an additional significant cohort as well as in other patient groups at risk of ARDS. Finally, our comparison of EVLWI between our patients in BALTI prevention cohort versus our oesophagectomy patients in the open label vitamin D replacement study is a potential limitation. However, the translational protocol of assessments and the two centres in which those assessments were performed was the same between the two studies. We are currently conducting a randomised placebo controlled trial to confirm these results due to finish recruitment in mid-2015, which should further address this question.^30^

Taken together, these data suggest that vitamin D deficiency is ubiquitous in patients with ARDS and relates to adverse outcome. In patients undergoing oesophagectomy, severe preoperative deficiency is associated with evidence of increased alveolar epithelial damage and EVLW accumulation as well as an increased risk of postoperative lung injury. Novel in vitro data further suggest a trophic and antiapoptotic role of physiologically relevant doses of 25(OH)D_3_ upon primary adult human alveolar epithelial cells. Finally, preoperative restoration of vitamin D levels in patients with oesophageal cancer who are at risk of ARDS resulted in significantly less accumulation of EVLW than in unsupplemented patients postoperatively.

In conclusion, we suggest that clinical strategies should be developed to replete vitamin D levels in patients at risk of ARDS and this approach might also have value as a treatment for established ARDS.

## Supplementary Material

Web supplement
