# Supplementary material for: Vitamin D deficiency contributes directly to the acute respiratory distress syndrome (ARDS)
Source: Thorax. 2015 May 1;70(7):617–24. doi: 10.1136/thoraxjnl-2014-206680 (PMC4484044; doi:10.1136/thoraxjnl-2014-206680)
Supplement: Web supplement [file thoraxjnl-2014-206680-s1.docx]

**Patient cohorts:**

Online Supplement:

ALI patients : Exclusion: age <18 years; participation in other intervention trials; severe obstructive airways disease requiring nebulised or intravenous β_2_ agonist; treatment with β blockers within 48 h; neutrophil count <0.3×10^9^/l; brainstem death; treatment withdrawal within 24 h; immunosuppression (glucocorticoids >20 mg prednisolone equivalent/day, chemotherapy or other immunosuppressive agents within 2 weeks); lobectomy/pneumonectomy; burns >40% body surface area; assent declined from the next of kin.

At risk patients: 57 consecutively recruited patients undergoing oesophagectomy via transthoracic oesophagectomy enrolled in the translational sub-study of BALTI-prevention. [^1^](#_ENREF_1) These patients have a ~25% overall risk of developing ALI post operatively.

A further 8 at risk patients undergoing oesophagectomy who had been vitamin D replaced with a median single bolus of 200,000 international units of cholecalciferol liquid 7-14 days prior to the day of operation were also studied from an open label vitamin D dosing study (ISRCTN6671978). Comparisons between the oesophagectomy patients are outlined in table 2.

Normal volunteers: 18 normal volunteers (10F:8M) mean age 46.9 (SD 14.5)

Lung tissue donors: 8 (6M:2F) mean age 63 (SEM 8.5) patients undergoing surgical lung resection for lung cancer with normal lung function were recruited using the Midlands Lung Tissue Collaborative.

**Methods:**

**Plasma preparation:**

Blood was collected from pre-sited arterial or central venous lines into 7ml Lithium Heparin vacutainer® tubes (Becton Dickinson Ltd, Oxford, UK). These were then transported on ice to the laboratory for processing and analysis. Whole blood was placed in a centrifuge pre-chilled to 4^o^c and spun at 560g for 10 minutes. The supernatant (plasma) was then aspirated and aliquoted into cryovials and stored at -80C

**PiCCO™ (Pulse Contour Cardiac Output Monitoring)**

Extravascular lung water (EVLW) was measured using the single-indicator transpulmonary thermodilution system (PiCCO-II™; Pulsion Medical Systems, Munich, Germany) as described previously. [^2^](#_ENREF_2) Briefly a 20-ml bolus of 0.9% saline at 4°C was injected via a central venous catheter into the right atrium. The thermodilution curve was recorded in the aorta and used to calculate EVLW and pulmonary blood volume. The average result from three 20-ml bolus injections was used for each measurement. EVLWI is the EVLW indexed by Ideal Body Weight, while the Pulmonary Vascular Permeability Index (PVPI) is the ratio of Pulmonary Blood Volume and EVLW. In our study, the CV for this system was less than 7% for all parameters.

**Vitamin D status**: 25(OH)D_3_ was measured by tandem mass spectronomy using appropriate DEQAS control. 1,25(OH)_2_D and Vitamin D binding protein were measured by ELISA.

**Multivariate analysis of At risk patient vitamin d levels and risk of lung injury.**

Unadjusted associations between 25(OH)D levels and ALI were estimated by bivariable logistic regression models.  Adjusted odds ratios (ORs) were estimated by multivariable logistic regression models with inclusion of covariate terms thought to plausibly associate with both 25(OH)D and ALI.  In this manner, we sought to create a parsimonious model that did not unnecessarily adjust for variables that do not affect bias or the causal relation between exposure and outcome. For the primary model ALI, specification of each continuous covariate (as a linear vs. categorical term) was adjudicated by the empiric association with the primary outcome using Akaike’s Information Criterion; overall model fit was assessed using the Hosmer Lemeshow test.

**Mouse Methods:** Wild-type (WT) C57Bl/6 mice were obtained from Harlan UK Limited, Oxford, UK and maintained at BMSU, Birmingham University, UK. The LPS challenge model was performed as described previously. ^15^ Briefly, mice are anesthetized and 50µg LPS (Sigma, UK) instilled by intra-tracheal (IT) route as a model of direct lung injury. In this model neutrophilic inflammation peaks at 48 hours. Mice were sacrificed and bronchoalveolar lavage (BAL) performed with two washes of 0.6ml PBS/EDTA (200nM) installations to determine the local effects on inflammation. BAL fluid was assessed for cellular inflammation by cell count and apoptotic cell number (flow cytometry), markers of epithelial damage BAL RAGE, protein permeability index (ratio of BAL: plasma protein) as well as cytokines by luminex array (R&D systems, UK). Oxygenation saturations and heart rate were measured by MouseOx II™. Once weaned, vitamin D deficiency was induced in WT pups by feeding them a vitamin D deficient chow (Harlan, USA) for 6 weeks pre-IT LPS. Results represent experiment on 18 wild-type mice fed normal chow and 12 wild-type mice fed the deficient chow. All experiments were performed in accordance with UK laws with approval of local animal ethics committee.

### Lung resection specimens

Resected lung specimens were immediately examined in the operating theatre by a member of the surgical team and a portion of specimen without macroscopic pathology and not required for a diagnostic purpose was passed to the research team. This sample was immediately immersed in sterile 0.9% saline in a sealed container and transported on ice to the laboratory for processing. The sample was measured and superficially washed with 0.9% saline immediately on arrival at the laboratory.

**ATII Cell isolation and culture:**

ATII cells were extracted according to methods described previously[^3^](#_ENREF_3). Average yields of ATII were 30.2 million cells per resection with a purity of 92%. Cells were tested for ATII cell phenotype by alkaline phosphatase staining, lysotracker lamellar body staining and by PCR expression of surfactant protein C—a type II cell marker with negative expression of aquaporin V (a type I cell marker) (data not shown). 0.5 Million cells were seeded onto 24 well plates and grown for 3 days in DCCM-1 (Biological Industries Ltd. Kibbutz Beit-Haemek, Israel) media supplemented with 10% fetal calf serum (FCS). Before stimulation cells were serum starved overnight (0.1% FCS) and stimulated in medium containing 0.1% FCS for 24 hours.

**Microarray analysis** Total RNA was extracted from control and treated cells using RNeasy kit (Qiagen). All RNA samples had high integrity and purity as assessed by NanoDrop ND1000 (NanoDrop Technologies). Sense-strand cDNA was generated from 200ng total RNA using Ambion® WT Expression Kit. Fragmentation and labeling was performed using the Affymetrix GeneChip WT terminal Labeling Kit. All kits were used according to the manufacturer’s recommendation. The cDNA was hybridized to Human Gene 1.0 ST arrays. The GeneChip Human Gene 1.0 ST Array offes whole-transcript coverage. Each of the 28,869 genes is represented on the array by approximately 26 probes spread across the full length of the gene, providing a more complete and more accurate picture of gene expression than 3’ based expression array designs. Probe cell intensity data (CEL) from Affymetrix (Santa Clara, California, USA) HuGene 1_0-ST microarrays were analyzed in the Affymetrix® Expression Console™ software with the default settings of the RMA-sketch workflow. Differentially expressed probe sets were identified using the limma package in Bioconductor project (http://www.bioconductor.org). For gene set enrichment analysis of we used the GOStat tool at http://gostat.wehi.edu.au. The cluster of co-expressed genes by the alveolar epithelial cells under the influence of 100nM Vitamin 25(OH)D3 was compared to the reference list i.e. set of all genes in the experiment. Using the binomial for each molecular function, biological process to obtain statistically over- and under-represented (p< 0.05) GOES categories.

**Wound repair, proliferation and cell death assays:** For viability and proliferation experiments, primary alveolar type II cells were used 24-48 h after extraction. For wound repair experiments, 0.5 million cells were seeded onto 24 well plates and grown for 4 days in DCCM (Troon Scientific, Troon, Perthshire, UK) media supplemented with 10% FCS. Wound repair assays were performed on confluent monolayers of ATII cells. Monolayers were wounded with a 2 mm mechanical wound, as described previously. [^4^](#_ENREF_4) The wounds were photographed under a microscope at 0 and 18 h and analysed with Scion image software

Bromodeoxyuridine (BRDU) incorporation of ATII cells was assessed using a colorimetric assay (Calbiochem, UK) according to the manufacturer’s instructions. FasL mediated cell death. Cells were pre-treated for 1 hour with 10ng/ml TNF-alpha and then 100 ng/ml of sFasL (R&D Systems, Abingdon, UK) was added for 24 hours. Cellular viability was measured using Cell Titre Aqueous (Sigma, Abingdon UK).

**Multivariate analysis of At risk patient vitamin d levels and risk of lung injury.**

Unadjusted associations between 25(OH)D levels and ALI were estimated by bivariable logistic regression models.  Adjusted odds ratios (ORs) were estimated by multivariable logistic regression models with inclusion of covariate terms thought to plausibly associate with both 25(OH)D and ALI.  In this manner, we sought to create a parsimonious model that did not unnecessarily adjust for variables that do not affect bias or the causal relation between exposure and outcome. For the primary model ALI, specification of each continuous covariate (as a linear vs. categorical term) was adjudicated by the empiric association with the primary outcome using Akaike’s Information Criterion; overall model fit was assessed using the Hosmer Lemeshow test.

**Microarray Results**

Table A List of 25 statistically significantly expressed up regulated genes in day 3 epithelial (Type-II like) cells treated with Vitamin 25(OH)D_3_ 100nM relative to untreated cells

| **Probe Set ID** | **Gene Symbol** | **Gene Description** | **P-value** | **Fold Change** |
| --- | --- | --- | --- | --- |
| 8067140 | CYP24A1 | Cytochrome P450 family 24 subfamily A polypeptide 1 | 0.000000 | 55.63 |
| 8044212 | SULT1C2 | Sulfotransferase family, cytosolic, 1C, member 2 | 0.000000 | 11.81 |
| 8157446 | ORM1 | Orosomucoid 1 | 0.003890 | 4.42 |
| 8157450 | ORM2 | Orosomucoid 2 | 0.000411 | 4.21 |
| 8091385 | CP | Ceruloplasmin (ferroxidase) | 0.000000 | 3.45 |
| 8103326 | FFG | Fibrinogen gamma chain | 0.004245 | 3.19 |
| 8137219 | C7orf29 | Chromosome 7 open reading frame 29 | 0.000259 | 3.08 |
| 8147132 | CA2 | Carbonic anhydrase II | 0.000000 | 2.98 |
| 8126303 | TREM1 | Triggering receptor expressed on myeloid cells 1 | 0.000342 | 2.91 |
| 8028955 | CYP2B7P1 | Cytochrome P450, family 2, subfamily B, polypeptide 7 pseudogene 1 | 0.000000 | 2.89 |
| 8026490 | UCA1 | Urothelial cancer associated 1 (non-protein coding) | 0.000029 | 2.70 |
| 8035095 | CYP4F11 | Cytochrome P450 family 4 subfamily F polypeptide 11 | 0.001395 | 2.63 |
| 8104074 | MTNR1A | Melatonin receptor 1A | 0.000799 | 2.61 |
| 8143499 | TRPV6 | Transient receptor potential cation channel, subfamily V member 6 | 0.002941 | 2.59 |
| 8028963 | CYP286 | Cytochrome P450 family 2 subfamily B polypeptide 6 | 0.000005 | 2.55 |
| 8146687 | ADHFE1 | Alcohol dehydrogenase, iron containing, 1 | 0.000003 | 2.44 |
| 7985555 | EFTUD1 | Elongation factor Tu GTP binding domain containing 1 | 0.000714 | 2.37 |
| 7914950 | CSF3R | Colony Stimulating factor 3 receptor (granulocyte) | 0.000001 | 2.37 |
| 8176133 | G6PD | Glucose-6-phosphate dehydrogenase | 0.000002 | 2.35 |
| 8042637 | DYSF | Dysferlin, limb girdle muscular dystrophy 28 (autosomal recessive) | 0.000004 | 2.34 |
| 8065905 | GDF5 | Growth differentiation factor 5 | 0.000108 | 2.18 |
| 8016646 | COL1A1 | Collagen type 1, alpha 1 | 0.000030 | 2.15 |
| 7957023 | LYZ | Lysozyme (renal amyloidosis) | 0.000001 | 2.14 |
| 7914342 | FABP3 | Fatty acid binding protein 3, muscle and heart (mammary-derived growth inhibitor) | 0.005937 | 2.12 |
| 8151191 | CPA6 | Carboxypeptidase A6 | 0.000001 | 2.05 |

Table B List of 25 statistically significantly expressed down regulated genes in day 3 epithelial (Type-II like) cells treated with Vitamin 25(OH)D_3_ 100nM relative to untreated cells after 24 hours.

| **Probe Set ID** | **Gene Symbol** | **Gene Description** | **P-Value** | **Fold Change** |
| --- | --- | --- | --- | --- |
| 8132118 | AQP1 | Aquaporin 1 (Colton blood group) | 0.00000 | -5.51 |
| 7901256 | CYP4B1 | Cytochrome P450, family 4, subfamily B polypeptide 1 | 0.00007 | -3.48 |
| 7934979 | ANKRD1 | Ankyrin repeat domain 1 (cardiac muscle) | 0.00000 | -2.81 |
| 8090823 | SLCO2A1 | Solute carrier organic anion transporter family, member 2A1 | 0.00053 | -2.27 |
| 7909318 | C4BPA | Complement component 4 binding protein alpha | 0.00016 | -2.22 |
| 8126729 | CLIC5 | Chloride intracellular channel 5 | 0.00252 | -2.17 |
| 8022655 | AQP4 | Aquaporin 4 | 0.00170 | -2.13 |
| 8139433 | MYO1G | Myosin 1G | 0.00001 | -2.11 |
| 8048864 | CCL20 | Chemokine (C-C motif) ligand 20 | 0.00000 | -2.10 |
| 8064894 | LRRN4 | Leucine rich repeat neuronal 4 | 0.00000 | -2.04 |
| 7940147 | FAM111B | Family with sequence similarity 111, member B | 0.00095 | -1.97 |
| 8083471 | SGEF | Src homology 3 domain-containing guanine nucleotide exchange factor | 0.00014 | -1.96 |
| 8152522 | ENPP2 | Ectonucleotide pyrophosphatase/phosphodiesterase 2 | 0.00831 | -1.94 |
| 8146092 | IDO1 | Indoleamine 2,3-dioxygenase 1 | 0.00003 | -1.93 |
| 8081488 | HHLA2 | HERV-H LTR-associating 2 | 0.00016 | -1.92 |
| 7927271 | FMO2 | Flavin containing monooxygenase 2 (non-functional) | 0.00074 | -1.91 |
| 7933750 | SLC16A9 | Solute carrier family 16, member 9 (monocarboxylic acid transporter 9) | 0.00011 | -1.85 |
| 7954810 | LRRK2 | Leucine-rich repeat kinase 2 | 0.00002 | -1.82 |
| 8162870 | BAAT | Bile acid Coenzyme A: amino acid N-acyltransferase (glycine N-choloyltransferase) | 0.00522 | -1.81 |
| 7982597 | THBS1 | Thrombospondin 1 | 0.00006 | -1.81 |
| 8165334 | CLIC3 | Chloride intracellular channel 3 | 0.00003 | -1.79 |
| 8052753 | GKN2 | Gastrokine 2 | 0.00361 | -1.77 |
| 8048870 | WDR69 | WD repeat domain 69 | 0.00000 | -1.75 |
| 7909306 | C4BPB | Complement component 4 binding protein beta | 0.00164 | -1.73 |
| 8082928 | CLDN18 | Claudin 18 | 0.00041 | -1.72 |

A heat map outlining the changed genes is illustrated below.

Expression heatmap of genes listed in table A and B of online supplement

References:

1. Perkins GD, Park D, Alderson D, Cooke MW, Gao F, Gates S, et al. The Beta Agonist Lung Injury TrIal (BALTI)--prevention trial protocol. *Trials* 2011;12:79.

2. Perkins GD, Nathani N, McAuley DF, Gao F, Thickett DR. In vitro and in vivo effects of salbutamol on neutrophil function in acute lung injury. *Thorax* 2007;62(1):36-42.

3. O'Kane CM, McKeown SW, Perkins GD, Bassford CR, Gao F, Thickett DR, et al. Salbutamol up-regulates matrix metalloproteinase-9 in the alveolar space in the acute respiratory distress syndrome. *Crit Care Med* 2009;37(7):2242-9.

4. Perkins GD, Gao F, Thickett DR. In vivo and in vitro effects of salbutamol on alveolar epithelial repair in acute lung injury. *Thorax* 2008;63(3):215-20.
